# Interfacing consciousness

**DOI:** 10.3389/fpsyg.2024.1429376

**Published:** 2024-07-15

**Authors:** Robert Prentner, Donald D. Hoffman

**Affiliations:** ^1^Institute of Humanities, ShanghaiTech University, Shanghai, China; ^2^Association for Mathematical Consciousness Science, Munich, Germany; ^3^Department of Cognitive Sciences, University of California, Irvine, Irvine, CA, United States

**Keywords:** conscious agent theory (CAT), interface theory of perception (ITP), conscious realism, AI consciousness, agency, computation, spacetime, simulation hypothesis

## Abstract

The current stage of consciousness science has reached an impasse. We blame the physicalist worldview for this and propose a new perspective to make progress on the problems of consciousness. Our perspective is rooted in the theory of conscious agents. We thereby stress the fundamentality of consciousness outside of spacetime, the importance of agency, and the mathematical character of the theory. For conscious agent theory (CAT) to achieve the status of a robust scientific framework, it needs to be integrated with a good explanation of perception and cognition. We argue that this role is played by the interface theory of perception (ITP), an evolutionary-based model of perception that has been previously formulated and defended by the authors. We are specifically interested in what this tells us about the possibility of AI consciousness and conclude with a somewhat counter-intuitive proposal: we live inside a simulation instantiated, not digitally, but in consciousness. Such a simulation is just an interface representation of the dynamics of conscious agents for a conscious agent. This paves the way for employing AI in consciousness science through customizing our interface.

## 1 The current impasse in the science of consciousness

There is a large consensus in the scientific community, according to which consciousness is somehow a product of information processing in the brain. There exist many different theories in the field (Signorelli et al., 2021), which have produced impressive new insights, such as discovering a range of candidates for the neural correlates of consciousness.^1^ However, these theories fail to also explain these correlations: why do they exist in the first place? To a physicist working on high-energy particle physics, it would surely seem very disappointing if the standard model were simply a list of correlations, say, between particle motions and detector values. Even if we were able to “furnish systematic correlations” (Seth and Bayne, 2022), this wouldn't provide much relief.

Yet, we believe there is a need to abandon the consensus view. We need new theories that actually do have the potential to explain, not just list or predict, these correlates. One such theory is the conscious agent theory (CAT; Hoffman and Prakash, 2014; Fields et al., 2018; Hoffman et al., 2023). CAT is presented as a theory of consciousness on its own terms, not a theory of consciousness as it arises from physical processes in the brain or elsewhere. One could forget everything one knew about physics, and still engage in CAT. But it would be a mistake to conclude from this that CAT is not a mathematically precise theory. On the contrary, it starts with a minimal but rigorously defined set of assumptions (Hoffman and Prakash, 2014):

1. Consciousness exists. We represent this by a (possibly infinite) set *X* of experiences. In Hoffman et al. (2023), this set was interpreted as an agent's potential to have experiences.
2. An agent could have this experience (e.g., seeing red), rather than that one (seeing green). The mathematical way to represent this is to say that the set of conscious experiences is measurable^2^ enabling us to state a probability to undergo any specific experience. An agent not only has the potential for conscious experiences but there are specific experiences that it undergoes at any given moment.

Unlike many other theories of consciousness, CAT takes agency as a fundamental ingredient. Only agents are conscious, and it is via their actions that they affect the world. Whereas experience reflects the private, first-personal aspect of consciousness, action consequences amount to its publicly observable, third-personal aspect. In CAT, this is formalized via conditional probabilities:

3. Consciousness makes a difference to the agent. There is a conditional probability that expresses how likely it is for a conscious agent to act in a certain way, given that it undergoes a specific prior experience.^3^ In CAT, this is called the “decision” of an agent. Consciousness also makes a difference to the world and its future perception by an agent. What is true for decisions, is also true for the execution of actions.

**Figure 1:**
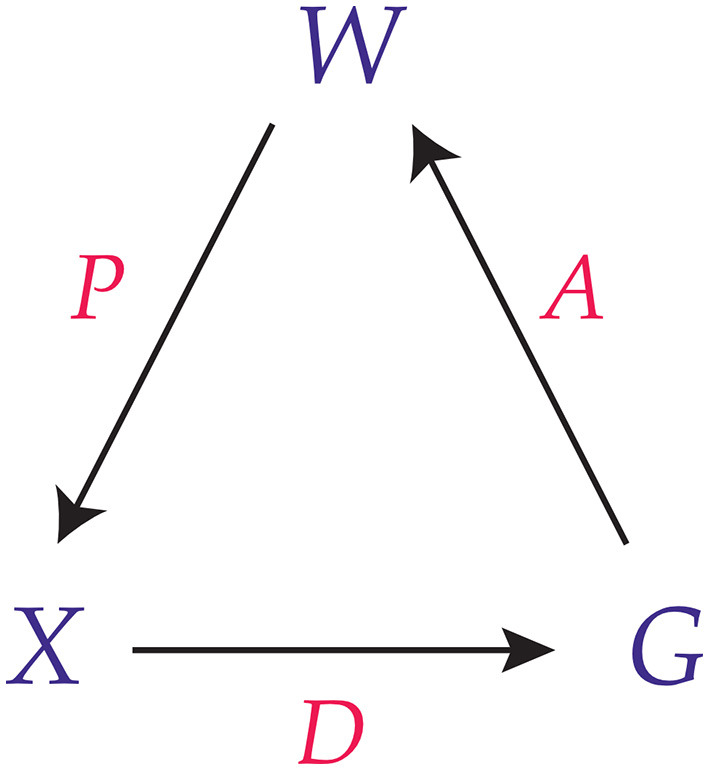
Conscious agent diagram, taken from Hoffman and Prakash (2014).

Together, this results in a tripartite structure that is shown in Figure 1. Other theories in consciousness studies use conditional probabilities as well, chiefly among them the “Integrated Information Theory of Consciousness” (Tononi et al., 2016; Albantakis et al., 2023). However, the difference is that integrated information theorists use conditional probabilities to specify a *physical substrate* of consciousness, whereas in CAT conditional probabilities are used to specify the dynamics of consciousness itself. Conditional probabilities have also long been suspected to play a crucial role in the computational approach to perception, e.g. according to a Bayesian model (Knill and Richards, 1996; Hoffman et al., 2015). Increasingly, this perspective gets adopted in predictive processing theories of consciousness too (Seth and Bayne, 2022). However, in CAT these are typically not seen as uncertainties about the perception of a physical world but as probabilistic elements inherent to consciousness.

It seems suggestive now to build networks of conscious agents that could account for many (or all) of the processes described by cognitive science (Fields et al., 2018). The idea here is not that consciousness is one more process built on top of many other supporting processes (such as learning, memory, representation, decision, etc.), but that consciousness provides the basis from which these processes emerge in the first place. More speculatively even, it has been proposed that physics itself arises from the combination and fusion of conscious agents (Hoffman et al., 2023).

We further believe that CAT has the resources to integrate a range of subjects from physics to AI. AI consciousness only starts to make sense once we abandon a physicalist worldview.

## 2 The interface theory of perception

On its own, the theory of conscious agents seems to be somewhat removed from the empirical day-to-day research in the scientific studies of cognitive (neuro)science. But to the avail of CAT, a recent proposal has been defended in the literature that provides an account of the formative processes underlying perception. The so-called interface theory of perception (ITP; Hoffman et al., 2015; Prentner, 2021) is deeply rooted in evolutionary theory and thus lies within the bounds of conventional scientific discourse. According to ITP, the things that we perceive (both objects and structures) arise as solutions to the problem of representing the world in a way that allows an agent to choose actions that increase its fitness. Fitness payoffs are agent-dependent values mapped from a domain that includes world states, the classes and states of agents, and their available action classes. Hence the relevant payoff functions are generically not homomorphic to the structure of the agent-independent world “out there” (Prakash et al., 2020). If I see an apple in front of me, what is the probability that there really is an apple in front of me, irrespective of the way I observe it? Almost certainly zero. If I see symmetries in the world, what is the probability that there really are symmetries in the world, irrespective of the way I can act on the objects I perceive? Almost certainly zero. If I perceive any structure at all, what is the probability that there really are those structures, irrespective of the way observers exist in the world? Almost certainly zero.

Our perceptions, do not mirror the world in any deeper sense apart from their consequences for fitness (Prakash et al., 2021). Rather than giving us an insight into the nature of reality, perception can be compared to a desktop interface. It allows an agent to successfully interact with its world, very much like dragging and dropping icons on a computer desktop allows us to move and delete files in the computer. This might sound similar to theories of the embodied mind (Chemero, 2009), sensori-motor contingency (O'Regan and Noë, 2001), or active inference (Clark, 2017; Parr et al., 2022). But other than those theories, ITP goes one step further and seeks to undermine our belief in physical objects that serve as embodiments, as substrates of sensory and motor processes, or as basis for inference.

Still, ITP leaves open an important question: if perception is an interface, what does it interface with?

## 3 Conscious realism

According to conscious realism, the whole universe can be represented as a network of conscious agents (Hoffman, 2008; Hoffman and Prakash, 2014). Hence, interfaces are used by conscious agents to represent networks of conscious agents — consciousness self-reflectively represents itself via interfaces. Thereby, agency is a fundamental concept. Many things can be said to exist in the universe. Among them are physical events in spacetime and subjective experiences. We propose that space and time can be derived from the network of conscious agents in terms of a representation by which agents, in order to act, make sense of the hyper-dimensional dynamics of consciousness.

But also our subjective experiences, such as our experience of the arrow of time can be recovered from an (unchanging) network of conscious agents. We typically think of our actions in terms of sequences of events in time. But time, in the theory of conscious agents, is a mere artifact of projection (Hoffman et al., 2023; Hoffman, 2024). One might note at this point that the intent of CAT is to re-conceptualize our view of the world and to serve as “theory of theories” that non-reductively links various areas of the natural world such as those studied by fundamental physics, evolution by natural selection, or cognitive science. Such a re-conceptualization is not only needed to explain the neural correlates of consciousness, namely as necessary correlations between a network of conscious agents and its (interface) representation, but to make sense of reality more generally.

Since CAT does not start by stipulating, from the outset, many of the typical features of our subjective experience such as selfhood or the experience of an arrow of time, it seems prudent to call the kind of minimal consciousness invoked by CAT a *non-dual*^4^ variety of consciousness. Indeed, as we saw in the basic definition of CAT reviewed in the first section, all that CAT is premised on is the idea that we have (a potentially infinite number of) experiences that can be individuated probabilistically and evolve in terms of conditional probabilities—if an agent were to experience *x* now, it will, with some positive probability, experience *y* later.^5^ At this stage, nothing yet has been said about the subjective/objective dichotomy, the objective structure of the world, or any quasi-axiomatization of subjective experience. By contrast, CAT is a relational theory from which one could recover different interface representations of the subjective experience of the agent in question (ideally with mathematical precision). But the experiences of many agents might be utterly unlike our own subjective experience.

## 4 Interfaces to consciousness

### 4.1 Spacetime

It is very unlikely that our species-specific interface bears any similarity with whatever lies underneath it. If the interface theory is right also on a fundamental level, the probability that this deeper reality is spatiotemporal in nature is close to zero. Although exotic at first sight, such a view seems to align well with recent findings from fundamental physics—at least if one lets go of the assumption that our classical (perceptual) model of reality is somehow approximating ground truth. Many physicists now believe that spacetime is not a fundamental entity. This is independent of the particular approaches endorsed by researchers such as Smolin (2001), Rovelli (2004), Gross (2005), or Arkani-Hamed (2010). Of course, it is still an open question what would replace spacetime, but all approaches agree that spacetime has to go eventually (see also Musser, 2017). Hoffman et al. (2023) advised to heed those physicists and link spacetime to the asymptotic dynamics of conscious agents, as it can be classified via the notion of a “decorated permutation.” Still, this is very counter-intuitive. After all, it certainly looks as if space and time are fundamentally real. But looks can be deceiving. And this is exactly what ITP tells us. Moreover, one might worry that the fact that we can do science of any kind presupposes space and time. But while the interface theory seems to imply that we should not take space and time as being there when no one looks, it still cautions us to take them seriously. And this dissolves the worry. ITP invites us to think of space and time as real for most practical purposes, but not simpliciter.

### 4.2 Agency and life

Consciousness is deeply linked to agency. Hence, one would perhaps expect to see the first glimpses of consciousness in living beings, which are — according to our present state of knowledge—the first instances of embodied agents that we can observe in the world. Yet, this merely reflects our ignorance of the fact that also the world underneath organisms might be rich in agency (a claim suggested by some interpretations of quantum mechanics such as QBism; von Bayer, 2016). Prebiotic agency normally stays invisible to us. But this could be a mere artifact of our (limited) interface. According to Nagel (1974): “if one travels too far down the phylogenetic tree, people gradually shed their faith that there is experience there at all.” We do not see any logical reason why this should stop at the living. However, what is different at the level of non-living beings is that it becomes harder to ascribe true agency there. It is in living beings that consciousness appears to us for the first time. But it appears in the form of *embodied* agents, not agency itself. Sometimes these embodiments give us more insights into consciousness (in the case of living beings), sometimes less (in the case of dead matter). Again, taking agency to be an exclusive property of living beings might be valid for most practical purposes, but not simpliciter.

### 4.3 Computation

In the theory of conscious agents, “computation” is not merely a concept that could be usefully employed to describe a certain empirical matter (as, for example, when we say “the brain computes”). It is inherent to the theory itself. At the moment, it is still unclear what non-computable functions a conscious agent network could implement. Yet, it is relatively straightforward to show that networks of conscious agents are computationally universal (Hoffman and Prakash, 2014), i.e., they could simulate other architectures known to be computationally universal (such as certain cellular automata or Turing machines). This fact was also exploited by Fields et al. (2018), who aimed to show how networks of conscious agents could implement various cognitive (read: computational) mechanisms. Given a purely formal definition of information (Cover and Thomas, 2006), CAT defines information processing in terms of (conditional) probabilities. In addition, conscious realism proposes that physics can be recovered from networks of conscious agents as an interface representation. Together, these claims would indicate that, contra Rolf Landauer's mantra “information is physical” (Landauer, 1999), the dynamics of consciousness fully accounts for a substantial notion of information processing. “Computation” would be one of many ways to describe the dynamics of consciousness as it appears on the interface of perception. Our claim is then that physics is information that comes from consciousness: IT from BIT from CIT.^6^

## 5 Consciousness and AI

### 5.1 A new paradigm

The question of whether artificial intelligence can become conscious currently gets much attention from scholars and media (Chalmers, 2022; Association for Mathematical Consciousness Science, 2023). According to the consensus view mentioned at the beginning of this article, one should expect computers to become conscious as soon as they implement the right computations (for example, mimicking the processes happening in our brains, Butlin et al., 2023). Yet, if consciousness is fundamental, it is inscrutable how computation could give rise to it. This appears to put us in a position that denies the possibility of AI consciousness. But this overlooks a crucial idea, which has to do with ITP. Accordingly, “computation” is just the name for an interface representation of the dynamics of consciousness. An interface hides and simplifies what lies beyond it. Yet, with the power of AI, we can custom-tailor our interface. Put differently, while we do not create consciousness in the process, we can use technology to help us get new insights into the (pre-existing) realm of conscious agents, similar to how we could use AI to get new insights in physics (Krenn et al., 2022). Yet, consciously experiencing these insights (including understanding them) is something that *we* need to do.

### 5.2 A simulation in consciousness

In his now-famous simulation argument, Bostrom (2003) proposed the following argument to show that we are “almost certainly living in a computer simulation”:

1. In the future, enormous computational resources will be available to a post-human society. One thing that members of this society will do is run computer simulations about their ancestors (i.e., us),
2. If you run the right computations, then the programs instantiating those computations will be conscious,
3. It is then (statistically) prudent to assume that we are just among those simulated beings, rather than being part of the original race that conceived the simulation.

Much has been written about the simulation argument. In particular, the claim of computationalism about consciousness strikes many as wrong, who are immersed in the scientific study of consciousness (Hoffman, 2019; Seth, 2021). A physicalist objection is that this wrongly assumes a strong notion of “substrate independence” (Prentner, 2017), the claim that the computations underlying consciousness can be instantiated in all kinds of substrates—no matter whether they are biological or artificial. But the objection can be easily countered by noting that advanced simulations will be fine-grained enough to simulate any physical system, and consciousness could then just run on such a “virtual machine.” By contrast, conscious realism accepts a variety of the simulation argument but with an important caveat: the simulation we are in is a simulation instantiated in consciousness! After all, consciousness—unlike a physical or biological system—is not a substrate that could itself be simulated. The reasons why the simulation argument (as stated by Bostrom and followers) is incorrect is not because it is not sufficiently physicalist, but because it is not sufficiently idealist.

## 6 Discussion

Conscious realism is the claim that the universe consists entirely of conscious agents. ITP says that we interact with this reality not directly but through a perceptual interface. These claims provide us with a new agenda for consciousness science in the future, resolving some challenges, but opening up others. Those challenges pertain to the nature of spacetime (it is not fundamental), agency (it is not limited to biological systems), and computation (it is not physical). Instead, CAT ultimately re-conceives these concepts as arising from the dynamics of conscious agents as we see them through an interface. In this light, to say that we live inside a simulation means that the simulation is what conscious agents are doing, as another conscious agent would perceive it. This paves the way for employing AI in consciousness science through customizing our perceptual interface.

## Data availability statement

The original contributions presented in the study are included in the article/supplementary material, further inquiries can be directed to the corresponding author.

## Author contributions

RP: Conceptualization, Writing – original draft, Writing – review & editing. DH: Conceptualization, Writing – review & editing.

## Footnotes

^1^Although the full story is not quite as straightforward, cf. Signorelli et al. (2021); Lepauvre and Melloni (2021).

^2^Technically, we need to endow the set *X* with a sigma-algebra X which defines possible “events” based on the underlying set. This sigma-algebra could be interpreted as our cognitive representation (Hoffman et al., 2023).

^3^More formally, this is done by defining a Markovian kernel over the set of experiences and actions of an agent.

^4^= non-objective but also non-subjective.

^5^Technically, as stated in Hoffman et al. (2023), this amounts to a “qualia-kernel” that would integrate over all possible actions and external states of the network.

^6^For the Indian doctrine of cittamātra see Westerhoff (2018).
